# Paulo Freire: Review of “The Pedagogy of the Oppressed”: 1st Edition, Penguin Random House UK, London, 2017

**DOI:** 10.1186/s12954-022-00605-9

**Published:** 2022-03-04

**Authors:** André Gomes

**Affiliations:** Release Legal Emergency and Drugs Service, Ltd, 61 Mansell Street, London, E1 8AN UK

**Keywords:** Pedagogy, Drug policy reform, Education, Oppression, Prohibition, Problem posing dialogue, Freire

## Abstract

Book review of “The Pedagogy of the Oppressed” by Paulo Freire.

Within the field of social justice, Paulo Freire requires no introduction. His philosophy-based pedagogy has greatly contributed to public education programmes in the 1960s Global South. Working first as a secondary school teacher and later as a university professor in Brazil, he developed a revolutionary pedagogic method that was highly successful in impoverished populations. He rose to prominence during João Goulart’s short-lived leftist government, working in the National Commission of Popular Culture until a military coup d’état in 1964. Forced into exile in Bolivia and Chile, Freire used his experiences in governance and teaching to write *Pedagogy of the Oppressed* in 1968.

While his work has been applied to different social justice areas like racial justice [1] and gay rights movements [2], there does not appear to be any academic output connecting Freire to the field of drug policy reform. Freire’s understanding of systems of oppression resounds greatly with the global regime of drug prohibition [3], a regime that dominates our understanding of drugs and prescribes what uses are valid (in many cases, legal) or not. Drugs have been so effectively portrayed as intrinsically evil, their use so dangerous and their users^1^ so immoral, that even those in the field struggle to emerge from this oppressive reality. Freire’s work devises a strategy to achieve freedom, understood here as liberty from the forces of oppression. This is a common goal across drug policy-concerned civil society within prohibition regimes.

This book review will focus on interpreting his critique of oppression through a drug policy lens in order to understand how valuable his insights are in challenging the dominant system of oppression in drug policy—prohibition. For this purpose, the oppressors are understood as the dominant class in society, typically the policy-makers; while the oppressed are seen as people who do not use illegal drugs but passively support the oppressive reality and people who use drugs, ranging from the most casual of users to the most problematic, including those struggling with drug dependency.

## Installing the oppressor versus oppressed through education

Freire perceives the current world as one entrenched in an ideology of oppression, defining oppression as: “Any situation in which ‘A’ objectively exploits ‘B’ or hinders his and her pursuit of self-affirmation as a responsible person… [interfering] with the individual’s… vocation to be more fully human”. In Freire’s book, the oppressors’ consciousness desires to “transform everything surrounding it into an object of its domination". The need to objectify humans is driven by their materialist nature, and the belief that “Humanity is a thing, and they possess it as an exclusive right”.

Conversely, the "oppressed are regarded as the pathology of the healthy society, which must therefore adjust these ‘incompetent and lazy’ folk to its own patterns by changing their mentality. These marginals need to be ‘integrated’, ‘incorporated’ into the healthy society that they have ‘forsaken’”.

Such rhetoric is common in drug-related public discourse; the dehumanisation of drug users transforms them into objects to surveil, discipline and punish, through either prison, stigma, coerced treatment, or anything in between. Specific to drug prohibition, this objectification is further codified by legally prohibiting (by criminalising) drug use. The oppressed are denied the chance to demonstrate their “responsibility”; the very act of trying drugs *even once* is deemed irresponsible, and those who use drugs are frequently described as immoral and damaging to society.

Freire highlights how the objectification of the oppressed in society, alongside uncritical models of education, results in the internalisation of oppression. The oppressed internalise “the image of the oppressor and [adopt] his guidelines”, and become fearful of freedom. The oppressed fatalistically accept their inferior position, and believe that the punishment, violence or condemnation they receive is deserved. The drug policy world suffers from this fear of freedom. People who use drugs internalise this oppressed identity, and come to see themselves as irresponsible and immoral, and society is unable to imagine different models for drug control. The oppressors’ objectification of the oppressed is then complete, as the latter internalise that they are not capable of the autonomy and responsibility that freedom requires: “So often do they hear that they are good for nothing…that they are sick, lazy, and unproductive—that in the end they become convinced of their own unfitness”.

Freire highlights how the oppressed come to engage in ‘necrophilic behaviour’: “the destruction of life—their own or that of their oppressed fellows”. He describes a peasant that, upon realising the futility of his actions against his oppressor, “shouts at his children, beats them, and despairs… the peasant gives vent to his sorrows by drinking”. This is one of the few, but noteworthy, references to substance use in *Pedagogy of the Oppressed*, as Freire demonstrates an understanding of how problematic drug use is a reaction to one’s reality under oppression, and how such cycle of oppression can breed generational violence.

This close reading of the methods through which oppression is established and reproduced unveils its alignment with many drug policy advocates’ critiques of prohibition. Knowledge presented as a universal ‘Truth’ constructs a reality that perpetuates prohibition and prevents the oppressed from achieving freedom, leaving them with few options beyond accepting their domination or wounding themselves and others in their despair.

## Challenging the oppression of drug prohibition

Only by contesting what is presented as the ‘Truth’, by imagining and building together a new version of what could be, can oppression be challenged and reversed. Liberation requires the reflective and total participation of the oppressed in the educational process. This includes reconfiguring student–teacher relationships into what Freire proposes are ‘student-teachers’ and ‘teacher-students’. These new roles acknowledge that both agents can learn from each other in a dialogical process, working together to build curricula grounded in the students’ realities and informed by the teachers’ critical knowledge. In a sentence, Freire proposes a *dialogical, problem-solving* model of education. The focus on *dialogue* ensures that a curricula is constituted by “the students’ view of the world”, whereby they identify the dominant themes (understood as ideas, values, or areas of social struggle) to be critically analysed. The teacher-students role is thus to re-present such themes as distinct problems, and jointly investigate how they could be solved. The emphasis on *problem-solving* ensures that the educational process is focused on creating targeted solutions to local issues relevant to the student population. This method can “surmount the situation of oppression… through transforming action [so] they can create a new situation, one which makes possible the pursuit of a fuller humanity”.

Effective communication is integral to build a pedagogy of the oppressed: it is only through dialogue that we can identify and embed their biggest problems in their education and create a plan to foster the growth of new possibilities. Freire says that “dialogue is the essence of revolutionary action”, dedicating almost an entire chapter to the key traits for true dialogue: *humility* about one's knowledge over others, *faith* in the oppressed's power to envision a new world, *hope* for a new future, *critical thinking* to inform one's actions, and most importantly, *love*. In a rare moment of overt tenderness in academia, Freire states that a profound love for the world and its people is essential to create the means for freedom: "If I do not love the world—if I do not love life—if I do not love people—I cannot enter into dialogue”. While prohibition is built on division, manipulation and domination, its opposition must be grounded in cooperation, organisation and unity to achieve true freedom. Just as Freire’s work is guided by revolutionary love, so too must drug policy reform. Revolutionary love is needed to re-humanise the oppressed, and to believe in their capacity to imagine, organise, and work towards a more humane and responsible existence with drugs.

Freire’s emphasis on dialogue and problem-solving is a valuable lesson for drug policy reform advocates. Grounding work in the experiences of the oppressed, from the most casual to the most problematic use of drugs, ensures that the future is neither created by distant elites, nor limited by ideology. The *Pedagogy of the Oppressed* encourages education to challenge entrenched ‘Truths’: what if we recognised that drug use does not always lead to addiction? What if we involved people who use drugs in policy-making processes? Its problem-posing approach to education prompts the exploration of options that cannot be fathomed through an oppressive system of prohibition, whose existence depends on dehumanising those it oppresses. A new system is needed.

These insights are particularly valuable for drug policy reform as they identify how important the classroom and other educational settings are in perpetuating the oppressive reality of prohibition. Most critically, it also identifies that perhaps reform alone is not the solution; the total reversal of an oppressive system may be the only way to prevent oppression and dehumanisation. A reformed pedagogical system, grounded in the lived reality of the oppressed, based on love, respectful dialogue and critical thinking, may just be what is needed to reject the moralistic ‘drug-free world’ and imagine a world of drug freedom.

## Data Availability

Not applicable.

## Footnotes

1 NB: people who use drugs will occasionally be referred to as “users” in this text; this is for brevity, rather than reducing people to labelling people based on their drug use. The author supports a move towards more people-centred descriptions of drug use.
